# Emotion and cognition: on the cognitive processing model of nostalgia

**DOI:** 10.3389/fpsyg.2024.1440536

**Published:** 2024-09-18

**Authors:** Shurong Cao

**Affiliations:** ^1^Institute of Foreign Literature and Culture, Guangdong University of Foreign Studies, Guangzhou, China; ^2^School of English Studies, Shanghai International Studies University, Shanghai, China

**Keywords:** nostalgia, emotion, cognition, timespace, social bonds, meaning

## Abstract

Nostalgia is a common emotional phenomenon, but its complexity has resulted in a limited understanding and interpretation. This study, grounded in emotional cognitive science, develops a cognitive processing model of nostalgia, encompassing its triggering mechanism, cognitive processes, and effects. The analysis indicates that nostalgia functions as an adaptive mechanism for individuals with deficiencies in belongingness and low-avoidance tendencies, helping them cope with disruptions in self-continuity by forging symbolic social bonds in a distant and idealized timespace. Essentially, nostalgia allows individuals to reconstruct alternate systems of meaning and value, which serve as references for defining self-worth and identity. This study advances the understanding of the complex cognitive processing involved in nostalgia and also provides an important reference for the study of complex emotions.

## 1 Introduction

Nostalgia is an emotional impulse that resides deep within the human soul. Traditionally, it refers to a longing for the past or one’s home (Pearsall, 1998; Sedikides and Wildschut, 2016). However, it was not until the 1970s and 1980s, with the advancement of emotional cognitive science, that nostalgia transcended its traditional classification and evolved into various theoretical dimensions. Over the past few decades, numerous studies have explored the cognitive representations of nostalgia (Johnson-Laird and Oatley, 1989; Wildschut et al., 2006; Zhou et al., 2012; Sedikides and Wildschut, 2016; Abakoumkin et al., 2017; De Brigard, 2018). Generally, nostalgia is no longer confined to the intuitive notions of “homesickness” or “longing for the past.” Instead, it now encompasses the existential dilemmas and adaptive mechanisms of modern individuals, thereby significantly expanding the scope and depth of nostalgia theory. Drawing on findings from emotional cognitive science, this paper decodes the triggering mechanism, cognitive processes, and effect of nostalgia, thus providing a solid theoretical foundation for understanding the nature and cognitive psychological processes of nostalgia.

## 2 Historical conceptions of nostalgia

Unlike other concepts, nostalgia has a distinct advantage in that its origins can be clearly traced (Landwehr, 2018). The term was first coined by a Swiss medical student named Johannes Hofer in 1688 to describe the extreme homesickness experienced by Swiss mercenaries stationed in distant regions. Hofer characterized nostalgia as a medical or neurological disease caused by a brain deficiency, which could be diagnosed through such symptoms as persistent thoughts about home, melancholy, insomnia, anorexia, weakness, anxiety, shortness of breath, and heart palpitations (Wilson, 2005; Landwehr, 2018). However, Hofer believed that this disease could be cured with proper treatment. Initially, it was widely believed that this disease was unique to the Swiss, but later studies demonstrated its prevalence in the French army and among soldiers in the American Civil War. While Hofer’s interpretation of nostalgia lacks sufficient scientific basis today, it is indisputable that he initiated the study of this concept.

Nostalgia was defined as a medical disease throughout the 18th and 19th centuries (Greenberg et al., 2004). However, since the late 19th and early 20th centuries, it began to be viewed as a “psychiatric or psychological disorder,” with major symptoms including anxiety, sadness, weakness, loss of appetite, insomnia, and fever (Greenberg et al., 2004, p. 201). As a variant of melancholia, nostalgia was seen as “a regressive manifestation associated with loss, grief, incomplete mourning, and, finally, depression” (Castelnuovo-Tedesco, 1980, p. 121). Additionally, it was found that soldiers, seamen, immigrants, and first-year boarding or university students were more prone to experiencing nostalgia (Cox, 1988).

With the development of communication networks and transportation, nostalgia is gradually being removed from the list of disorders associated with distance. Many academics have begun to define nostalgia as an emotion based on homesickness and a longing for the past, for instance, Davis (1979, p. 5) claimed that “it is much more likely to be classed with such familiar emotions as love, jealousy, and fear than with such “conditions” as melancholia, obsessive compulsion, or claustrophobia.” While nostalgia is originally a private experience triggered by homesickness, nowadays it has evolved into a collective emotional experience due to commercialization, marketing, and politicization. For instance, the populist political movements around the world have achieved remarkable electoral success in the past decade by exploiting nostalgia appeals to an idealized past, of which Donald Trump’s 2016 presidential campaign slogan “Make America Great Again” is a very prominent and successful example. Fundamentally, this slogan conjured up images of a bygone golden age that needed to be reclaimed in order to make the nation prosperous again. Even those of Americans who have not personally experienced the glorious past, may find themselves looking back to the previous time with fondness, hoping to restore a sense of collective continuity. As Wilson (2005, p. 31) has noted, “nostalgia thus operates in both a public and private domain.” It is important to understand that the cognition of nostalgia should not fall into an either/or cognitive trap. We need to consider both the individual as well as the social orientation of nostalgia, as it presents a kind of “cultural embeddedness” within the “individual presentation.” This also means that we must analyze it within specific historical, cultural, and social contexts.

Emotion theorists generally agree on labeling nostalgia as an emotion. However, there is still controversy over its emotional signature (Abakoumkin and Green, 2021). The term “nostalgia” is derived from two Greek words, *nostos* and *algos*, meaning “return home” and “suffering” respectively. Initially, nostalgia was regarded as a negative emotion, akin to the pain caused by homesickness (Hertz, 1990; Holbrook, 1993). Although nostalgia may evoke pleasant memories of the past, it also highlights the irreversibility of those times. Furthermore, nostalgia has a negative aspect because individuals immersed in the past may find it difficult to respond effectively to the present.

Yet, some theorists emphasize the positive aspects of nostalgia (Davis, 1979; Kaplan, 1987; Gabriel, 1993; Holak and Havlena, 1998; Chaplin, 2000). For instance, Davis (1979, p. 14) suggested that nostalgia “is infused with imputations of past beauty, pleasure, joy, satisfaction, goodness, happiness, and love.” Similarly, Kaplan (1987, p. 462) described nostalgia as “a warm feeling about the past, a past that is imbued with happy memories, pleasure, and joy.”

Neither positivity nor negativity alone can capture the full essence of nostalgia. Consequently, some scholars emphasized the bittersweet flavor of nostalgia (Johnson-Laird and Oatley, 1989; Barrett et al., 2010). According to Castelnuovo-Tedesco (1980, p. 122), nostalgic recollection is typically “bittersweet.” It is sweet because the nostalgic object brought pleasure, which is amplified by the idealization process inherent in nostalgia. However, it is also bitter because the object of nostalgia is forever lost, and even in its original context, it was fraught with contradictions and disappointments. Additionally, Holak and Havlena (1998) highlighted the hybrid nature of nostalgia. Although nostalgia encompasses both positive and negative aspects, the key point is how it “regulates negative states, soothing emotional conflicts” (Yang et al., 2022, p. 1133). The regulation mechanism and cognitive reappraisal of nostalgia have become central issues in current nostalgia research.

The perception of nostalgia as an emotion persisted until the end of the 20th century. The development of cognitive neuroscience in the 1970s led scholars to realize that emotions, particularly complex ones, are intricate derivatives based on individual experiences and are always intertwined with people’s motivations and cognitive judgments (Weinberg et al., 2013). The emergence of this theoretical perspective shifted theorists’ attention to the cognitive nature of nostalgia.

It is generally recognized that our inner lives are dominated by two kinds of phenomena: thoughts (formally known as cognition) and feelings (formally called emotions). Since neither can be directly observed by others, both are relatively subjective or, at least, particular to an individual (Chalmers, 2007). Despite this, “there are strong, but often conflicting theoretical statements on cognition and emotion in the psychology literature” (Robinson et al., 2013, p. 4). For a long time, cognition and emotion have been seen as two very different modes of information processing: a rational system and an emotionally driven experiential system, respectively (Epstein, 1994). Indeed, since ancient Greek times, it has been prevalent in Western philosophy that cognition is a source of rationality and proper behavior, whereas emotion causes many problems for people. In fact, this emotion-cognition dichotomy permeates many Western literary works. Shakespeare’s tragedies, for example, often illustrate the conflict between emotion and reason inherent in human beings. Although Renaissance humanism affirms the rationality of human emotions, the boundlessness of eroticism tends to swing to the other extreme, leading to the proliferation of carnal and materialistic desires. This ambivalence constitutes, to a certain extent, the central conflict in Shakespeare’s plays.

The traditional dichotomy between emotion and cognition has been challenged by advancements in neuroscience in the late 20th century. Historically, it was believed that the eyes, ears, and other sensory organs transmit signals to the thalamus, which then relays them to the cortical neurons (the thinking brain) and the amygdala (the emotional brain). In this view, the amygdala, part of the primitive brain and the limbic system, serves as the neural basis of emotions and is responsible for the rapid generation of desire, rage, madness, and extreme happiness. Conversely, the neocortex is primarily involved in higher cognitive functions, such as sensory cognition, spatial cognition, language, motor command generation, and logical reasoning.

However, empirical studies conducted by three prominent affective neuroscientists in the 1990s questioned this traditional understanding. LeDoux (1998) discovered that the emotional brain, particularly the amygdala, can react more quickly and decisively to physical threats than cortical pathways. Panksepp (1998) demonstrated that as long as the emotional areas of the brain function properly, cortical lesions in animals often result in behaviors that are more meaningful from a functional biological perspective. Damasio (1994) extended his research to human patients with brain damage and found that damage to the parts of the brain controlling emotions, even when cognitive capacities remain intact, can lead to problematic decision-making or impulsive and reckless interpersonal behaviors. These studies do not suggest that emotion is more functional than cognition but indicate that emotion and cognition are not completely isolated entities. Instead, they frequently interact with each other.

The development of neuroscience has motivated theorists to focus on the cognitive nature of emotion. Simply put, emotion is not a random and irrational emotional catharsis but rather a complex emotional-cognitive schema. This theoretical breakthrough has opened up new directions for contemporary nostalgia studies. Since nostalgia encompasses emotion (mixed emotions), cognition (e.g., memory), and, to some extent, behavior, it transcends mere sentimentality and takes on a rich cognitive dimension. Cavanaugh (1989, p. 603) described nostalgia as “a cognitive attempt to recapture a time when life was good, safe, secure, and contented.” As a complex emotion, nostalgia “depends on a propositional content reflecting high-level cognitive evaluation giving rise to them” (Johnson-Laird and Oatley, 1989, p. 103). Therefore, it is reasonable to assert that “nostalgia involves a whole host of cognitions and emotions” (Wilson, 2005, p. 25).

## 3 The cognitive reconstruction of nostalgia

Nostalgia is a basic human sentiment; almost everyone experiences being triggered by an old photo or object. For some people, this emotion dissipates quickly, while for others, it lingers, especially when they use nostalgia as a mechanism to interact with their environment over time. This can develop into an emotional-cognitive schema for coping with complex realities.

### 3.1 What triggers nostalgia?

If nostalgia is the result of complex cognitive processing, the first question we need to answer is: What triggers nostalgia? As Lazarus (1982, p. 1019) has stated, “cognitive activity is a necessary as well as sufficient condition of emotion,” to be more specific, “emotion results from an evaluative perception of a relationship (actual, imagined, or anticipated) between a person and the environment” (Lazarus, 1982, p. 1023). It is reasonable, therefore, to infer that nostalgia starts from a negative cognitive evaluation of the environment (Shaw and Chase, 1989). For example, older people are more likely to experience nostalgia as they realize their time left is limited, which can create a sense of anxiety and panic. More importantly, a notable decline in social engagement and participation is frequently observed in elderly populations, leading to social isolation, loneliness, and dissatisfaction with social relationships (Victor et al., 2005). These factors altogether make older people prone to longing for the “good old days” and tighter social bonds. Moreover, nostalgia is intertwined with the social environment at the collective level. Davis (1979, p. 34) suggested that nostalgia often “occurs in the context of present fears, discontent, anxieties, or uncertainties, even though they may not be in the forefront of awareness.” Generally, contemporary scholars tend to link nostalgia with modernity. Although modernity has brought about some liberties and improvements, its ills have swept us away from all forms of traditional social order. Boym (2008, p. XVI), for instance, described nostalgia as “a symptom of our age.” According to her, traditional society is characterized by wholeness, intimacy, and transcendental worldview, whereas modern society emphasizes the objectification of human relationships through capitalism and the increasing bureaucratization of daily life.

Many current researchers typically attribute nostalgia to a sense of loss caused by unfavorable environment or reality. However, this explanation fails to fully elucidate the unique conditions under which nostalgia arises, as adverse situations can lead to various forms of identity crises and different coping strategies. In this regard, the research conducted by Wildschut et al. (2006) is instructive in addressing this interpretive gap. Their empirical studies demonstrated that nostalgia is elicited by deficiencies in belongingness. People have the fundamental need to belong, and those with strong and effective social bonds tend to perform better (Baumeister and Leary, 2017). However, since social relationships are dynamic, the deterioration and even dissolution of significant and valuable social bonds in some transitional periods can leave individuals feeling adrift and isolated (Colson, 1971). Consequently, individuals deploy a series of compensatory mechanisms or strategies to fulfill their belongingness needs. Gardner et al. (2013) categorized these social strategies into direct and indirect strategies. Direct strategies involve attempts to address belongingness deficiencies by revising existing relationships or forming new relational structures. Indirect strategies come into play when suitable interaction partners are not available in reality, prompting individuals to construct symbolic social bonds. Nostalgia belongs to the latter category. By rekindling meaningful relationships in the imagination, nostalgia strengthens social connectedness and provides individuals with positive relational knowledge structures (Baldwin et al., 1996).

The aforementioned studies mostly focus on the influence of the environment on individuals while to some extent ignoring the significance of individual variable. Simply put, although maintaining a sense of social belonging is vital for psychological well-being, not all people would seek relationships as a source of psychological comfort (Abeyta et al., 2019, p. 200). In other words, not everyone experiencing a crisis of belonging would resort to nostalgia; a person’s attachment type is also a crucial factor in the generation of nostalgia, as it influences whether or not a person would seek the support of interpersonal bonds in the face of distress.

There are three proposed attachment types, namely secure attachment, anxious attachment, and avoidant attachment. According to attachment theory, attachment-related anxiety reflects the degree to which the self is seen as worthy of love and support, whereas attachment-related avoidance reflects the extent to which others are seen as responsive to one’s distress (Brennan et al., 1998; Wildschut et al., 2010). And in recent years, an increasing amount of empirical research (Abeyta et al., 2015a; Abakoumkin et al., 2017; Abeyta et al., 2019) has demonstrated how attachment-related avoidance shapes nostalgia. Avoidant attachment is primarily related to emotional neglect during childhood, indicating that their need for intimacy was not adequately satisfied. Therefore, “individuals who rely on avoidant strategies minimize their attachment needs due to expectations of rebuff or rejection by romantic partners, especially in times of stress” (Cassidy and Shaver, 1999, p. 490). However, avoidant attachment can be further divided into two types: high-avoidance and low-avoidance. High-avoidance individuals perceive others as “unavailable or unresponsive” (Wildschut et al., 2010, p. 574), hence they are assumed to avoid intimacy and relational closeness and strive for personal success and competence (Shaver and Mikulincer, 2002; Abeyta et al., 2015b; Abeyta et al., 2019). In contrast, low-avoidance individuals view others as responsive and inwardly desire acceptance from others, making them more likely to harness nostalgia as a source of social connectedness (Wildschut et al., 2010, p. 518). Similarly, the studies conducted by Abeyta et al. (2015a), Abakoumkin et al. (2017), and Abeyta et al. (2019) also confirmed that nostalgia is more frequently used among those scoring low in attachment-related avoidance. In summary, nostalgia is triggered under the following three conditions: deficiencies in belonging caused by an unfavorable environment, a tendency towards low-avoidance, and an inability to realize the reconstruction of social bonds in reality.

### 3.2 How does nostalgia work?

After identifying the trigger mechanism of nostalgia, another critical question arises: How does nostalgia work? Before delving into the cognitive processes involved, it is essential to clarify the objects of nostalgia. Expanding upon traditional theories, the intentional object is “autobiographical recollection,” specifically memories of one’s hometown (De Brigard, 2018, p. 6). It is important to note that “home” extends beyond the literal house or street one was raised upon; it encompasses a broad spectrum of potential referents such as the school attended or the playground frequented. Moreover, “home” is not restricted to specific locations; it also refers to childhood experiences, old friends, food, customs, traditions, and more (McCann, 1941). Recent studies by Batcho (1995) and Hepper et al. (2014) further illustrated that people may feel nostalgic about a wide array of things beyond just places.

The autobiographical dimension of nostalgia is well-known. However, it cannot be denied that people may feel nostalgic about events that are not autobiographical. Simply put, nostalgic sentiments can be directed towards periods or events that we did not directly experience. A new term, “anemoia,” has been coined to describe this variant of nostalgia. The *Dictionary of Obscure Sorrows* defines anemoia as nostalgia for a time you have never known. As De Brigard (2018, p. 3) argued “nostalgic events involve the mental simulation of possible events that may or may not have happened in one’s own past,” suggesting that nostalgia is a blend of memory and imagination.

Indeed, many researchers have meticulously explored the complexity and diversity of nostalgic objects. Nawas and Platt (1965) proposed three types of nostalgia: past-oriented, present-oriented, and future-oriented. The first type revolves around the idea of a “homing instinct,” such as returning to one’s hometown, childhood, or even to the womb. The second type views nostalgia as a reaction to an individual’s unsuccessful adaptation to their present environment. The last type links nostalgia to uncertainty about goals and pessimism regarding future prospects. According to Boym (2008), the object of nostalgia is so elusive that it is difficult to pinpoint exactly what people yearn for.

Typically, the aforementioned statements are accompanied by the assumption that the intentional objects of nostalgia pertain to different times and spaces far removed from the present. In terms of time, nostalgia can refer to “the past as imagined, as idealized through memory and desire” (Hutcheon, 2000, p. 195), but it can also look toward the future. Futurist nostalgia constructs a spiritual fortress against the injustices of reality while simultaneously depicting a vision of social reform and progress (Spender, 1965). Spatially, the objects of nostalgia are no longer confined to one’s hometown but rather point to any spiritual home far from reality, which can include Elsewhere, or even Nowhere. It should be noted that the idealized home, whether in the past or present, has never existed in reality and “is not made of individual memories but of collective projections and rational delusions” (Boym, 2008, p. 43). In a sense, certain types of nostalgic longing can be associated with “political and social manipulations,” which means that nostalgia is sometimes employed to justify national or political goals (Clewell, 2013, p. 260). To some extent, the widespread political appeal of “Make America Great Again” (MAGA) put forward by Donald Trump, the former president of the United States, represents the utilization of nostalgia in political rhetoric. Essentially, this slogan serves as a manifestation of national nostalgia, assisting political actors to mobilize support and realize political purposes by conjuring up vague images of bygone golden ages.

Further evidence of the diversity of nostalgic objects becomes apparent when we consider the question: What do nostalgic individuals truly long for? Perhaps, it is not space or time that is the true object of nostalgia. Wilson (2005, p. 18) argued that “what people nostalgize for is the things which symbolize what people wish for.” In other words, nostalgia reveals what we value and deem worthwhile and important. Wilson’s answer is somewhat abstract. Given that nostalgia stems from deficiencies in belongingness that cannot be resolved in reality, it is reasonable to assume that what nostalgic individuals long for is a symbolic sense of belonging in different timespaces, which constitutes the ultimate object of nostalgia.

If nostalgia is a longing to recapture a sense of belonging, then what cognitive mechanisms should be employed to achieve symbolic compensation? I propose that the most prominent cognitive strategies are distancing and idealization.

Firstly, distancing is necessary for nostalgia. Commenting on Romantic nostalgia, Boym (2008, p. 13) stated that the nostalgic object “must be beyond the present space of experience, somewhere in the twilight of the past or on the island of utopia where time has happily stopped.” Hutcheon (2000, p. 195) shared a similar view, claiming that “nostalgic distancing sanitizes as it selects, making the past feel complete, stable, coherent--in other words, making it very unlike the present.” By distancing oneself from reality, largely through imagination, the nostalgic subject can defend against the sense of loss and anxiety caused by an unfavorable environment. It should be made clear from the outset that as nostalgic people are often trapped in an unfavorable reality, imagination is therefore needed to construct a harmonious coexistence with the environment in a faraway timespace. Thus, distancing is more a product of imagination than action, and the idealized spacetime constructed is more of a spiritual home than a real one.

Secondly, another cognitive strategy associated with nostalgia is idealization. Idealization is believed to be a fundamental element of nostalgia (Santesso, 2006). As Howard (2012, p. 643) observed, “nostalgia imaginatively projects desirable features onto the past, rather than represents qualities which the past possessed.”

As a typical defense mechanism, idealization is a minor image-distorting defense whereby an individual deals with emotional conflicts or internal and external stressors by attributing exaggerated positive qualities to oneself or others (American Psychiatric Association, 1994; Perry, 1990). Here, it is important to distinguish between nostalgic idealization and narcissistic idealization. Narcissists, in order to alleviate feelings of insignificance, tend to idealize their strengths or outstanding qualities, sometimes even portraying themselves as a superhuman figure. In contrast, the objects of nostalgic idealization are the others. This distinction perhaps lies in the fact that nostalgia aims at the reconstruction of belongingness and self-continuity, rather than glorifying oneself. Specifically, nostalgic idealization consists of two dimensions: the idealization of heterogeneous timespace and social bonds.

Speaking of nostalgia, it originally signifies an overwhelming longing for one’s home. However, the concept of home was both spatial and temporal, and often unattainable, which led to nostalgia evolving into a longing for an idealized memory of home rather than an actual place. Those afflicted with nostalgia can project their longing onto an imagined timespace, such as a divine nature, perfect utopia, or a golden past, by removing some of the complex factors in reality to make the imagined environment simple, ordered, and controllable. Therefore, nostalgia is not “simple fond remembrance, but the imagination of an idealized timespace” (Tinsley, 2020, p. 2330).

Moreover, the idealized timespace is frequently “constructed (and then experienced emotionally) in conjunction with the present, which, in turn, is constructed as complicated, contaminated, anarchic, difficult, ugly, and confrontational” (Hutcheon, 2000, p. 195). Take nostalgic literature as an example: Mark Twain’s *The Adventures of Huckleberry Finn* is a typical novel of nostalgia in which Twain contrasts the American “Gilded Age” with the idealized timespace composed of a teenager, a runaway slave, and a small boat. Despite the ugliness and shackles of the real world, the protagonists could fully enjoy friendship, innocence, and freedom in their small but idealized world.

Nostalgia often arises from a deficiency in belongingness, leading individuals to yearn for harmonious, warm, and reliable social bonds. Unlike the idealized home envisioned by the Romantics, the beautiful spiritual home for the nostalgic is always “peopled” (Hertz, 1990, p. 195), forming various kinds of harmonious and warm relationships. These intimate relationships may have been experienced by individuals, but more often than not, they are constructed through idealization. The social nature of human beings dictates that they cannot construct their identity in isolation; rather, they perceive and define themselves within various social relations. It is believed that “human fitness is enhanced with the maintenance of successful relationships with others” (Heine et al., 2006, p. 96). Therefore, people are naturally attracted to intimate and long-lasting relationships, through which they can obtain social support and emotional comfort. Highly contextualized as nostalgic imagination is, the most commonly-constructed social bonds longed for by the nostalgic include kinship, friendship, romantic love, and so on. Jesmyn Ward, a prominent contemporary African American writer, portrays the deep love between grandfather and grandson in her novel *Sing, Unburied, Sing*. Ernest Hemingway’s *The Sun Also Rises* highlights romantic love separated by life and death. The depiction of idealized social bonds is also frequently seen in Chinese literature. *The Journey to the West*, one of China’s four classics, constructs an ideal relationship between master and disciple.

The need to belong is evident not only in people’s desires to form interpersonal relationships but also in their desires to belong to a social group. When people in similar situations or with similar temperaments gather together, they develop a sense of connectedness and belonging. This nostalgic strategy is fully represented in popular American sitcoms such as *Friends* and *The Big Bang Theory*. In literature, it is also alluded to in Toni Morrison’s *Song of Solomon* and Edith Wharton’s *The Age of Innocence*, among others. Interestingly, in modern society, as interpersonal relationships become increasingly alienated, it becomes more difficult to construct and maintain traditionally idealized bonds. Consequently, some unconventional relationships, such as human-pet relationships and human-clone relationships, are gaining popularity. Movies like *Paddington Bear* and *Blade Runner* 2024 can be categorized as this category.

According to those findings, I can safely propose that nostalgia functions as a complex cognitive schema, with distancing and idealization as two major cognitive strategies. These strategies help individuals construct symbolic social bonds in an idealized timespace in their nostalgic reverie, “presumably in an attempt to compensate for their weakened feelings of belongingness” (Heine et al., 2006, p. 96).

### 3.3 What does nostalgia achieve?

Nostalgia is often considered a compensatory cognitive strategy, which has led to various criticisms. Some theorists have associated it with conservatism or, even worse, regression. For instance, Su (2005, p. 2) argued that being nostalgic is being “out of touch,” reactionary, and even xenophobic. However, reconstructing symbolic social bonds in an idealized timespace is not merely a retreat from reality but rather a positive search for meaning.

Individuals have an inherent need for meaning. Many philosophers, such as Kierkegaard (1997), Heidegger (1996), and Camus (1955), have argued that life is a never-ending pursuit of meaning. But what exactly is meaning? According to Heine et al. (2006, p. 90), “meaning connects elements of the self: thoughts, behaviors, desires, attributes, abilities, roles, and autobiographical memories.” They further explained that “any disruption to one’s meaning framework, particularly with respect one’s relations with the external world, creates a sense of urgency to construct another relational framework” (Heine et al., 2006, p. 98). Although Heine’s team does not explicitly clarify the content of meaning, it can be reasonably deduced that the meaning is closely linked to a sense of belonging, security, stability, and so on. For example, individuals in unhappy marriages might choose to have more children to compensate for their deteriorating feelings of belongingness within the marriage.

Unlike the reconstruction of meaning in reality, nostalgia primarily involves the symbolic production of meaning. Specifically, through the cognitive processes of memory and imagination, individuals experiencing nostalgia connect elements related to themselves in a way they expect within an imagined timespace, thus creating an alternate framework of meaning. In this sense, nostalgia provides “a sanctuary of meaning—a place where one feels she knows herself; where identity has safe harbor” (Wilson, 2005, p. 10).

Furthermore, the meanings embedded in the idealized timespace and social bonds constitute the value labels by which individuals define themselves. This contributes to constructing an ideal self that is unattainable in the current unfavorable environment. Therefore, “the object of nostalgia (and hope) is neither place nor time, but the subject, the self that was and is yet to be” (Bradbury, 2012, p. 342).

## 4 Conclusion

Nostalgia, as a fundamental and prevalent emotion, is not merely an instinctive physical and mental reaction of human beings but a social cognitive phenomenon formed through the interaction of complex objective factors and multiple cognitive constructions. This interaction also leads to the diverse forms of nostalgia. As illustrated by the above analysis, nostalgia is a complex, systematic cognitive project that relies on the creative interplay between nostalgic individuals and their unfavorable environments. It requires the coordination and cooperation of different cognitive processes, including distancing, idealization, and alternate meaning and value frameworks, to achieve emotional compensation. Any weakness or flaws in these links may affect the cognitive efficacy of nostalgia. Overall, I hope this study helps to reveal many unknown cognitive processes behind nostalgia and aids researchers in understanding the complex and diverse constructions of nostalgia in different fields.

## Data Availability

The original contributions presented in the study are included in the article/supplementary material, further inquiries can be directed to the corresponding author.
